# Knowledge, Awareness, and Perceptions of Artificial Eyelash Use and Associated Ocular Side Effects Among Palestinian Females: A Cross-Sectional Study

**DOI:** 10.7759/cureus.86753

**Published:** 2025-06-25

**Authors:** Ithar M Beshtawi, Dana Walweel, Warda Sakhel, Aseel Shashtare

**Affiliations:** 1 Department of Optometry, Faculty of Medicine and Allied Medical Sciences, An-Najah National University, Nablus, PSE

**Keywords:** artificial eyelashes, awareness, cosmetics, palestinian females, side effects

## Abstract

Background

Despite the ocular side effects associated with artificial eyelashes, their usage is increasing among females globally. However, there is a lack of research on this topic among females in the Arab world. This study investigates the awareness and knowledge of artificial eyelash use and its associated issues among Palestinian females.

Methods

This study utilized a descriptive cross-sectional design and involved 499 female participants, with a mean age of 26.1 years (±8.2). Participants completed a self-administered questionnaire that collected data on demographics, awareness, reasons for use, usage behavior, and associated ocular adverse effects related to artificial eyelashes.

Results

A total of 91.8% (n=458) of participants were aware of artificial eyelashes, with 53.3% (n=266) reporting prior usage. There was a significant association between awareness and both age (p=0.00) and education level (p=0.00). The highest usage was observed in females aged 15-24 (n=184, 69.2%), with a significant proportion reporting lifetime use of only once (n=219, 82.3%) and usage limited to one day (n=202, 75.9%). This usage was significantly influenced by educational level (p=0.00). The main reason for use was aesthetic (n=195, 73.3%). Social media was the primary source of information regarding artificial eyelashes (n=310, 62.1%) and their associated side effects (n=103, 38.7%), while only a small percentage (n=25, 9.4%) sought information from health workers. The most frequently reported side effects were heavy eyelids (n=40, 15%), shadowing in vision (n=38, 14.3%), and tearing and itching (n=32, 12%). The frequency and duration of use showed a significant association with the occurrence of symptoms (p=0.04).

Conclusion

The use of artificial eyelashes is prevalent among Palestinian females for cosmetic enhancement. A significant proportion of participants reported ocular side effects, highlighting the need to enhance awareness among young females through the dissemination of evidence-based clinical information via social media.

## Introduction

The eyes are considered a focal point of interest when individuals first encounter others. Specifically, females often attempt to enhance the attractiveness of their eyes through the use of ocular cosmetics [1], kohl [2], or artificial eyelashes that are longer and thicker [3-6]. The latter involves the attachment of nylon or natural fibers using adhesive glue that contains latex, phthalates, and formaldehyde, a known carcinogen, which can induce allergic and toxic reactions in the conjunctiva, resulting in keratoconjunctivitis [7,8]. Prior studies, whether experimental [9,10] or survey-based [3-6,11], have investigated the ocular surface side effects of artificial eyelash use. The Tear Film and Ocular Surface Society (TFOS) lifestyle report [12] highlights the effects of cosmetics and artificial lashes on the ocular surface, noting symptoms such as itching, redness, pain, tearing, discharge, and misdirected lashes. Additionally, a few studies [3,7,10,11,13] reported changes in the eyelid, including heaviness, swelling, and irritation, alongside incomplete or reduced blinking. Dry eye disease symptoms were also reported in some studies among users of artificial eyelashes [9,10,14]. More advanced complications may arise due to inadequate hygiene and insufficient eyelid cleansing while wearing lashes, leading to the accumulation of dust, sweat, and potential pathogens [7,12,15]. These factors can result in gland blockages, keratoconjunctivitis [7], or keratitis [15].

Although ocular symptoms are associated with artificial eyelash use, their acceptance and popularity remain widespread among young females in various parts of the world. A national survey [3] conducted in Japan revealed a prevalence of 10.3% among 2,000 participants. In Saudi Arabia [16], a study found a prevalence of 35.3% among 207 participants aged 18 to 45 who had used artificial eyelashes at least once in their lifetime. A higher prevalence was observed in Nigeria [4], where 38.7% of 310 participants aged 16 to 52 reported using false eyelashes. In a subsequent study [6], this figure increased to 67.7% among 2,052 individuals aged 16 to 35. Additionally, a study [5] in Korea reported an even higher prevalence, with 84.9% of 448 adult women over the age of 20 having used artificial eyelashes.

The use of artificial eyelashes is a popular trend among women in the Arab world, particularly during social events and gatherings. However, scientific literature on the usage of artificial eyelashes and awareness of the associated ocular side effects remains limited. This study aims to investigate the awareness, knowledge, and potential eye problems related to artificial eyelash use among Palestinian females. The findings may help develop public health strategies, guide educational initiatives on safe cosmetic use, and enrich the limited regional data on the relationship between beauty practices and ocular health.

## Materials and methods

A descriptive cross-sectional design was used in this study. Data were collected through a self-administered, structured questionnaire developed by the researchers, aligned with prior studies [4,6] examining female knowledge and awareness regarding the use of false eyelashes. The questionnaire was distributed electronically via Google Forms. It underwent validation through a pilot study involving 15 females who use false eyelashes and was subsequently revised based on feedback from two professionals in the field. The questionnaire consisted of three primary sections: the initial section gathered basic characteristics of the study participants, including age, gender, educational level, familiarity with false eyelashes, and sources of information regarding false eyelashes. The second part inquired about awareness and experience with false eyelashes, including the age at first use, reasons for usage, frequency of use, duration of wear, use of semi-permanent lashes (i.e., for up to one month), and motivations for repeated use. The final section inquired about any related ocular symptoms (itching, casting of a shadow in vision, burning sensation, discharge, tearing/watering, red eye, eyelid swelling, pain, foreign body sensation, misdirected lashes, heavy eyelids, lashes falling out, and eyelid styes) reported by participants while using false eyelashes. The questionnaire was administered from March to June 2025 to 499 Palestinian females in beauty salons, shops, eye care centers, and their homes. Any participants who did not provide consent were excluded from the study.

This research adhered to the principles outlined in the Declaration of Helsinki. Ethical approval was obtained from the Institutional Review Board (IRB) committee at An-Najah National University prior to the initiation of the study (AAMS. Jan. 2025/2). Informed consent was obtained from each participant, and confidentiality was maintained.

The Statistical Package for the Social Sciences version 20.0 (SPSS Inc., Chicago, IL, USA) was utilized for data entry and statistical analysis. Based on a medium-sized effect and an alpha level of 0.05, this study had 80% or higher power to identify false eyelash extension awareness among Palestinian females, assuming data from 266 participants were analyzed. Descriptive analysis and simple frequencies (expressed in percentages) were used to describe the participants’ demographics and knowledge and awareness of false eyelash use. Non-parametric statistics were applied as the data were not normally distributed. Pearson's chi-square test was used to assess associations among categorical variables. A p-value of ≤ 0.05 with a 95% confidence level was deemed statistically significant.

## Results

A total of 499 females participated in this study, with 266 reporting active use of eyelash extensions, resulting in a usage rate of 53.3%. Participants' ages ranged from 15 to 54 years, with a mean age of 26.1 ± 8.2 years. Table 1 presents the distribution of participants across various age groups, educational levels, and familiarity with false eyelashes. It shows that 45.1% (n=225) were aged between 20 and 24 years, 75.8% (n=378) had attained a university education, and 91.8% (n=458) were aware of false eyelashes. A chi-square test was conducted to examine the association between demographic factors and awareness of eyelash use. A statistically significant association was found between participants' age group (p=0.00) and their educational level (p=0.00) with awareness of eyelash use. Additionally, 62.1% (n=310) of participants reported that their information regarding false eyelashes came from social media, while 20.7% (n=103) cited friends or relatives as their source. The analysis revealed no statistically significant relationship between age group and educational level concerning the source of information (p=0.17).

**Table 1:** Sociodemographic characteristics and awareness of false eyelashes among study participants (N = 499). Data are presented as frequency (N) and percentage (%).

Variable	Frequency (N)	Percentage (%)
Age group		
15-19	69	13.80%
20-24	225	45.10%
25-29	53	10.60%
30-34	41	8.20%
35-39	32	6.40%
40-44	33	6.60%
45-49	20	4.10%
50-54	26	5.20%
Level of education		
Illiterate	2	0.40%
Primary school education	7	1.40%
Middle school education	13	2.60%
Secondary school education	65	13.00%
University education	378	75.80%
Postgraduate studies	34	6.80%
Awareness of false eyelashes		
Yes	458	91.80%
No	41	8.20%
Sources of information about false eyelashes		
Friends and relatives	103	20.70%
Social media	310	62.10%
Books and magazines	45	9.00%
Not aware	41	8.20%
Previous use of false eyelashes		
Yes	266	53.30%
No	233	46.70%

Table 2 presents data on participants' awareness and use of false eyelashes. A total of 266 participants reported using false eyelashes, with the majority (n=184, 69.2%) first trying them between the ages of 15 and 24. About 73.3% (n=195) stated that they used false eyelashes for cosmetic enhancement, showing a significant association with educational level (p=0.00). Additionally, 65.8% (n=175) reported using them repeatedly for social events, with this behavior significantly influenced by both age (p=0.02) and educational level (p=0.03). In this sample, 82.3% (n=219) indicated that they had used false eyelashes at least once in their lifetime, while 75.9% (n=202) reported using them for only one day. This usage pattern was also significantly influenced by educational level (p=0.00). A smaller proportion, 29.7% (n=79), reported having tried semi-permanent eyelashes (i.e., for up to one month) at least once, whereas 51.9% (n=138) indicated they had never used semi-permanent lashes. The use of semi-permanent eyelashes was significantly associated with educational level (p=0.00).

**Table 2:** Participant awareness and use of false eyelashes (N = 266). Data are presented as frequency (N) and percentage (%). Significant at p < 0.05.

Variable	Frequency (N)	Percentage (%)	Age		Education	
			χ²(df)	p-value	χ²(df)	p-value
Age at first use of false eyelashes						
15-19	103	38.70%	χ²(49)=408.18	0.14	χ²(35)=77.07	0.7
20-24	81	30.50%				
25-29	24	9.00%				
30-34	19	7.10%				
35-39	16	6.00%				
40-44	17	6.40%				
45-49	5	1.90%				
50-54	1	0.40%				
Reasons for using false eyelashes						
Cosmetic/beauty	195	73.30%	χ²(63)=61.58	0.52	χ²(45)=80.01	0.00*
Peer influence	38	14.30%				
Mark of social class	26	9.80%				
Others (curiosity, etc)	7	2.60%				
Frequency of using false eyelashes						
Once a week	6	2.30%	χ²(21)=23.66	0.31	χ²(15)=61.76	0.00*
Once every two weeks	8	3.00%				
Once a month	33	12.40%				
Tried only once so far	219	82.30%				
Frequency of using semi-permanent false eyelashes						
Never used	138	51.90%	χ²(21)=23.01	0.34	χ²(15)=66.98	0.00*
Once	79	29.70%				
Twice	18	6.80%				
Three times	13	4.80%				
More than four times	18	6.80%				
Duration of wearing false eyelashes before removal						
One day only	202	75.90%	χ²(21)=20.02	0.52	χ²(15)=33.42	0.00*
Less than a week	26	9.80%				
One to two weeks	15	5.60%				
Up to a full month	23	8.70%				
Reasons for repeated use of false eyelashes						
Social events	175	65.80%	χ²(77)=103.11	0.02*	χ²(55)=67.41	0.03*
Routine lifestyle	31	11.60%				
Demand/request from partner	4	1.50%				
Others (photo shoots, birthdays)	56	21.10%				
Main source of information about the side effects of using false eyelashes						
Social media	103	38.70%	χ²(91)=85.54	0.64	χ²(65)=36.87	0.99
Friends and relatives	64	24.10%				
Health workers	25	9.40%				
Personal experience	74	27.80%				

**Figure 1:**
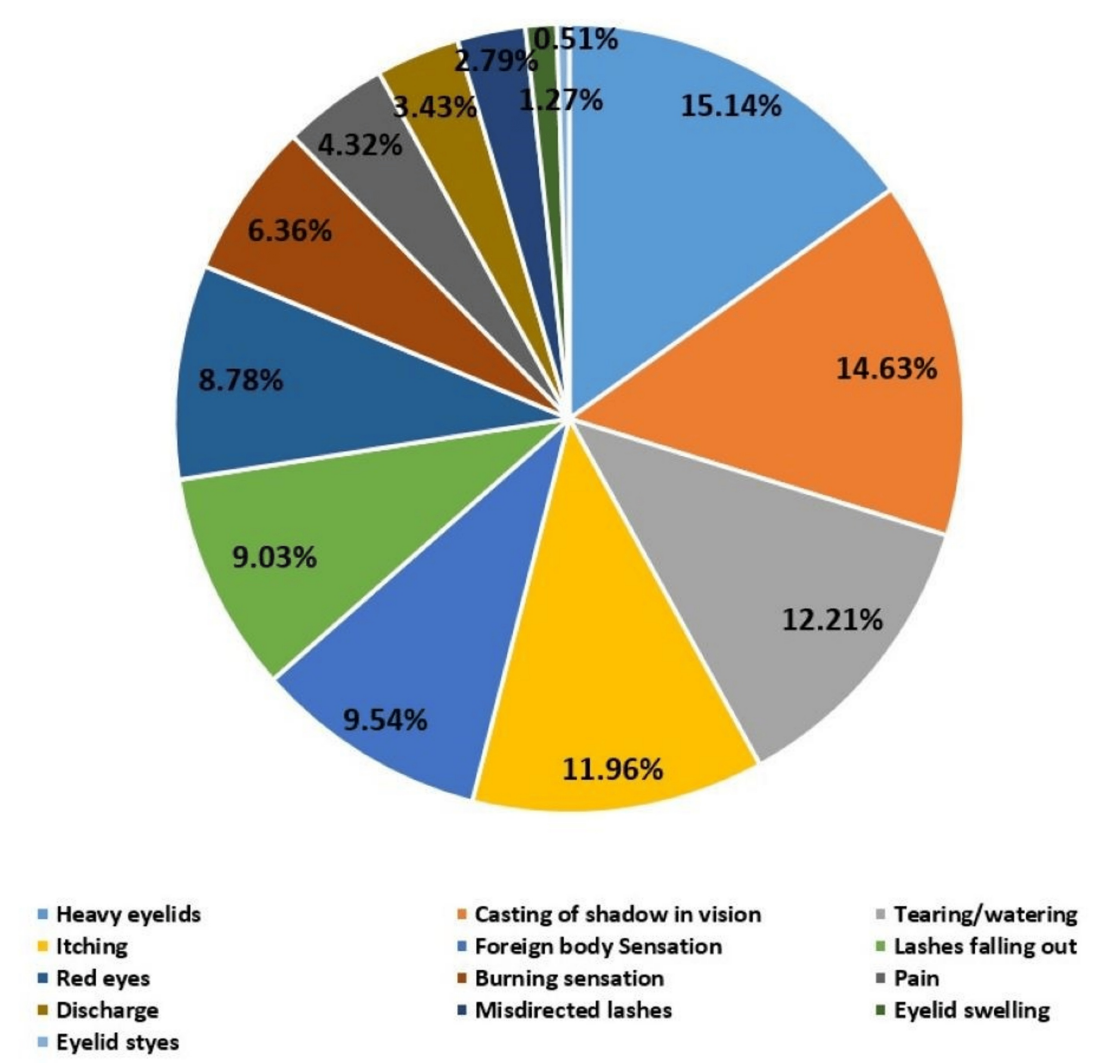
Ocular symptoms reported by participants (N=266). Data are presented as percentages (%).

The majority of participants reported obtaining information about the side effects of false eyelashes from social media (38.7%, n=103), while the least reported source was health workers (9.4%, n=25), as shown in Table 2. Among artificial eyelash users, 178 participants (66.9%) reported experiencing at least one ocular side effect, with a mean of 2.9 ± 2.4 symptoms. A chi-square test revealed a statistically significant association between the number of reported ocular symptoms and both the frequency (p=0.04) and duration (p=0.04) of artificial eyelash use. Figure 1 illustrates the most commonly reported side effects: heavy eyelids were noted by 15.0% (n=40), shadowing in vision by 14.3% (n=38), and tearing by 12.0% (n=32). Additionally, 1.1% (n=3) reported eyelid swelling, and 0.75% (n=2) reported styes, making these the least frequently reported symptoms.

## Discussion

The use of artificial eyelashes has become increasingly popular among young women worldwide [4-7] as a cosmetic enhancer [4-6,13], despite emerging evidence of their potential ocular adverse effects [6,7,9-17]. The use of artificial eyelashes among Arab females appears to be on the rise; yet, there is a paucity of research examining the prevalence and frequency of their usage in this demographic. Artificial eyelashes are frequently observed in both public environments and ophthalmic clinics, where users report related ocular problems. Despite this growing trend, awareness of use and associated problems remains inadequately investigated in the Arab world. This study reports findings on the awareness and knowledge of the use of artificial eyelashes among Palestinian females and the associated potential ocular problems.

This study indicated that 91.8% (n=458) of participants were familiar with eyelash extensions. This finding is consistent with a prior study that indicated a 99.7% awareness level among their participants [6]. A statistically significant association was found between the awareness of artificial eyelashes and both the participants' age group (p=0.00) and educational level (p=0.00). This might be explained by the fact that younger individuals and those with higher levels of education are more likely to seek health-related information, which may lead to increased awareness. The majority of participants (n=310, 62.1%) identified social media as their primary source of information regarding false eyelashes, followed by 20.7% (n=103) who referenced friends or relatives. No statistically significant relationship was observed between age or educational level and the source of information (p=0.17). This suggests that social media is extensively accessed across demographic groups for information regarding artificial eyelashes, regardless of age or educational background. The widespread use of social media as an information source raises concerns regarding the accuracy and reliability of the related content. This emphasizes the necessity of publishing evidence-based information via social media to reduce misinformation and promote safe practices.

In the current study, over half of the participants (n=499, 53.3%) had used eyelash extensions, indicating a prevalent trend in Palestine. This figure exceeds findings from Japan (10.3%) [3], Saudi Arabia (35.3%) [16], and Nigeria (38.7%) [4], yet is lower than a recent study in Nigeria [6] with a larger sample focused on the 16-35 age group (67.7%) and findings from Korea (84.9%) [5]. The variation in sample size and age range across these studies may have contributed to the differing results. Furthermore, cultural perceptions of beauty and religious norms may also influence the differing usage rates observed among countries.

The highest usage of artificial eyelashes in this study was observed among participants aged 15-24 years (n=184, 69.2%). This finding is consistent with prior research conducted in Japan [3], Korea [5], and Nigeria [4,6], where similar age groups were identified as the most frequent users of artificial eyelashes. Young females at the threshold of adulthood are often more conscious of their physical appearance, susceptible to emerging beauty trends, and influenced by social media and beauty influencers.

The current study indicates that 73.3% (n=195) of participants cited the enhancement of cosmetics and beauty as the primary motivation for using artificial eyelashes. This finding highlights the significance of beauty and aesthetics in the decision to use artificial eyelashes. Cosmetic enhancement demonstrated a statistically significant association with educational level (p=0.00), suggesting that individuals with different educational backgrounds may have distinct perceptions of cosmetic practices. Peer influence was identified as the secondary motivator, reported by 14.3% (n=38) of participants, indicating that social factors play a role in usage, though not as the main driver. These results align with findings from other studies, which also identified cosmetic enhancement as the primary reason for artificial eyelash use, though with varying percentages: 39.1% [5], 56.1% [4], 65.4% [6], and 81.6% [13]. This variation may stem from differences in cultural beauty standards, levels of social influence, and exposure to beauty trends across countries.

The majority of participants (n=219, 82.3%) in this study reported using artificial eyelashes only once in their lifetime, and 75.9% (n=202) indicated that their usage was limited to a single day. This occasional usage was significantly associated with educational level (p=0.00), indicating the influence of education on cosmetic habits. Additionally, 65.8% (n=175) of participants identified social events as the main factor influencing their decision for repeated use, which was significantly influenced by age and educational level (p=0.02 and p=0.03, respectively). The findings indicate that Palestinian females typically reserve the use of artificial eyelashes for significant personal occasions, such as weddings, rather than using them as a regular practice. This infrequent use of artificial eyelashes corresponds with results from a prior study [6], which reported that 43.1% of participants had used artificial eyelashes only once. Additionally, social events were also identified as the primary driver for repeated use [6]. Other studies have indicated a higher frequency of usage among participants, with 69.2% [13] and 65.5% [4] reporting repeated use of more than three times. The observed differences may indicate the impact of cultural and economic factors on daily cosmetic practices across countries. Regarding semi-permanent eyelash extensions, half of the participants in the present study (n=138, 51.9%) reported that they had never used them, while 29.7% (n=79) reported using them only once. The limited usage can be attributed to the elevated costs associated with semi-permanent eyelash application and the prolonged time needed for professional application. Temporary lashes can be applied swiftly without requiring a visit to a beauty salon.

Social media served as the main source of information about the adverse effects associated with false eyelashes for 38.7% (n=103) of participants in the study. Furthermore, 27.8% (n=74) acquired their information through personal experience, whereas 24.1% relied on insights from friends and relatives. Only 9.4% (n=25) indicated that they received such information from health workers. In a prior study [6], it was found that 37.9% of participants gathered their information from personal experience, followed by friends or relatives at 32.9%, social media at 19.5%, and health workers at 9.5%. These figures raise concerns about the accuracy of information regarding the adverse effects of artificial eyelashes, as users primarily rely on social media, which may downplay the risks associated with cosmetic products. Furthermore, personal experience suggests that users become aware of adverse effects only after experiencing them, rather than through proactive education. The limited reliance on information from healthcare professionals results in missed opportunities for early intervention. A total of 66.9% (n=178) of participants indicated the experience of at least one ocular symptom. This figure is lower than that reported in previous studies conducted in Nigeria (73.3%) [4] and Ghana (97.5%) [13], but much higher than that reported in Japan (26.8%) [3]. A statistically significant association (p=0.04) was found between the number of symptoms reported and both the frequency and duration of false eyelash use, indicating that more frequent and prolonged use of false eyelashes may increase the risk of associated ocular problems. This finding supports a prior study [11] that identified a significant association between the usage pattern of artificial eyelashes and ocular side effects.

The most predominant ocular adverse effects found in this study were heavy eyelids, casting of shadows in vision, tearing, and itching. Other symptoms identified included foreign body sensation, lashes falling out, redness, burning, pain, discharge, misdirected lashes, eyelid swelling, and styes. Numerous prior studies [3,4,6,10,11,15] have identified itching as a significant ocular side effect associated with the use of artificial eyelashes. This symptom may arise from the adhesive glue used to attach the lashes, which contains substances such as latex, phthalates, and formaldehyde [7,8]. These substances are known to provoke allergic and toxic reactions in the conjunctiva, leading to itching [7]. Previous studies have reported additional related symptoms, including heavy eyelids [3,4,11,13], burning [11,13], redness [3,4,11], foreign body sensation [10,11], pain [3,4,11], tearing [13], and lashes falling out [11]. Failure to address these issues may result in more serious complications, including blepharitis and keratoconjunctivitis [7]. Despite the associated side effects, females may persist in the use of artificial eyelashes [13]. Therefore, it is essential to educate users on the potential impact on eye health to reduce the frequency of artificial eyelash application.

The results of this study rely on self-reported data rather than clinical examination, which may introduce recall bias. Additionally, the study did not include diagnostic clinical tests to assess changes to the ocular surface, such as alterations to the palpebral conjunctiva that may be affected by the use of false eyelashes. Future research should address these limitations by incorporating diagnostic clinical assessments alongside self-reported data and by implementing a longitudinal study design with an expanded sample size across various countries in the Arab world.

## Conclusions

In conclusion, this study presented useful insights into the awareness and knowledge, as well as the associated ocular symptoms, related to artificial eyelash use among Palestinian females. The results revealed a high level of awareness and prevalence of artificial lash use, although such use is infrequent and mainly driven by cosmetic enhancement for social gatherings. A high percentage of participants reported experiencing ocular side effects, especially with frequent and prolonged use. Social media was considered the primary source of information about the use of artificial eyelashes and their potential side effects, while healthcare professionals’ involvement was minimal in this context. These results indicate the necessity to enhance awareness about the safe use of artificial eyelashes and related ocular problems via social media, particularly among young females.

## Appendices

Appendix 1

This questionnaire aims to assess the awareness, opinions, and usage of artificial eyelashes among females in Palestine. It includes questions about your background, your knowledge and experience with false eyelashes, and any eye-related symptoms you may have experienced.

Participation is completely voluntary, and all responses will be kept confidential.

**Table 3:** Sociodemographic characteristics and responses on awareness, perception, and use of false eyelashes – questionnaire.

False Eyelash Use Questionnaire
Do you agree to take part in this study? (Please select one option)
Yes
No
Which of the following is your age group? (Please select one option)
15-19
20-24
25-29
30-34
35-39
40-44
45-49
50-54
What is the highest level of education you have completed? (Please select one option)
Illiterate
Primary School Education
Middle School Education
Secondary School Education
University Education
Postgraduate Studies
Are you aware of false eyelashes? (Please select one option)
Yes
No
What are your sources of information about false eyelashes? (You may select more than one option)
Friends and relatives
Social Media
Books and Magazines
Not aware
Have you ever used false eyelashes? (Please select one option)
Yes
No
At what age did you first use false eyelashes? (Please select one option)
15-19
20-24
25-29
30-34
35-39
40-44
45-49
50-54
What are your reasons for using false eyelashes? (You may select more than one option)
Cosmetic/beauty
Peer influence
Mark of social class
Others (curiosity, etc)
How often do you use false eyelashes (i.e occasional, daily lashes)? (Please select one option)
Once a week
Once every two weeks
Once a month
Tried only once so far
How many times have you used semi-permanent false eyelashes (i.e., for up to one month)? (Please select one option)
Never used
Once
Twice
Three times
More than four times
How long do you typically wear false eyelashes before removing them? (Please select one option)
One day only
Less than a week
One to two weeks
Up to a full month
What are your reasons for repeatedly using false eyelashes? (You may select more than one option)
Social events
Routine lifestyle
Demand/request from partner
Others (photoshoots, birthdays)
What is your main source of information about the side effects of using false eyelashes? (Please select one option)
Social media
Friends and relatives
Health workers
Personal experience
Which of the following eye symptoms have you experienced related to the use of false eyelashes? (You may select more than one option)
Heavy eyelids
Casting of shadow in vision
Tearing/watering
Itching
Foreign body Sensation
Lashes falling out
Red eyes
Burning sensation
Pain
Discharge
Misdirected lashes
Eyelid swelling
Eyelid styes
